# Gastric bypass alters the dynamics and metabolic effects of insulin and proinsulin secretion

**DOI:** 10.1111/j.1464-5491.2007.02240.x

**Published:** 2007-11

**Authors:** H-E. Johansson, M. Öhrvall, A. Haenni, M. Sundbom, B. Edén Engström, F. A. Karlsson, B. Zethelius

**Affiliations:** Department of Public Health and Caring Sciences/Geriatrics, Uppsala University Hospital Uppsala, Sweden; * Department of Surgical Sciences, Uppsala University Hospital Uppsala, Sweden; † Department of Medical Sciences, Uppsala University Hospital Uppsala, Sweden

**Keywords:** gastric by-pass, insulin, obesity, proinsulin

## Abstract

**Aims:**

Hyperproinsulinaemia is associated with obesity and is a risk factor for Type 2 diabetes. We explored the dynamics of proinsulin and insulin and postprandial effects on glucose and lipids in subjects who had undergone gastric bypass (GBP) surgery compared with morbidly obese (MO) subjects and normal weight control subjects (NW).

**Methods:**

Subjects free from diabetes were recruited: 10 previously MO subjects [body mass index (BMI)±sd, 34.8±6.2kg/m^2^] who had undergone GBP surgery, 10 MO subjects (BMI 44±3.1kg/m^2^) and 12 NW control subjects (BMI 23.2±2.4kg/m^2^). After an overnight fast, a standard meal (2400 kJ) was ingested and glucose, proinsulin, insulin free fatty acids and triglycerides were determined up to 180 min.

**Results:**

Fasting proinsulin was similar in the GBP group and NW control subjects, but threefold increased in MO subjects (*P <* 0.05). Postprandial AUC for glucose was similar in the three groups and AUC for proinsulin was high in MO, intermediate in the GBP group and lowest in NW control subjects (*P* for trend = 0.020). Postprandial proinsulin at 60 min was similar in the GBP group and MO subjects and twofold higher than in NW control subjects. Postprandial proinsulin at 180 min was normal in the GBP group, but fivefold increased in MO subjects (*P =* 0.008). Insulin increased rapidly at 30 min in the GBP group and was normal at 90 min, whereas insulin was still increased at 90–180 min in the MO subjects (*P <* 0.001).

**Conclusions:**

MO subjects, free from diabetes, have elevated proinsulin concentrations in the fasting as well as the postprandial phase. After GBP surgery markedly lower fasting and postprandial proinsulin concentrations were observed, although BMI was higher compared with NW control subjects.

## Introduction

Obesity is associated with insulin resistance [1], fasting hyperinsulinaemia [2] and fasting hyperproinsulinaemia [3]. In addition to insulin resistance [4], the hyperproinsulinaemia, which reflects pancreatic B-cell strain, is associated with increased risk of Type 2 diabetes mellitus (T2DM) in men [5,6] and women [7], and with coronary heart disease (CHD) morbidity [8–10] and mortality [10] in men. Elevated proinsulin predicts conversion to T2DM independent of peripheral insulin resistance and early insulin response during a glucose tolerance test [5,6]. Moderate weight loss is associated with improved insulin sensitivity and lowered hyperinsulinaemia [11] and a reduced incidence of T2DM [12,13]. Pronounced weight loss after gastric bypass (GBP) surgery in subjects with morbid obesity is associated with a marked improvement in insulin sensitivity [14], lower fasting hyperinsulinaemia [15], lower postprandial hyperinsulinaemia [16] and a markedly reduced conversion to T2DM over 10 years of follow-up [17]. GBP surgery is also known to cause rapid transit of nutrients to the bowel, with rapid elevations of glucose and insulin. To our knowledge, there is no report on postprandial proinsulin and insulin and their interrelationship in morbid obesity after GBP surgery. The aim of the present study was to investigate fasting and postprandial proinsulin and insulin responses, effects on glucose, free fatty acids (FFA) and triglycerides (TG) in previously morbidly obese subjects with GBP-induced weight loss, in comparison with subjects with morbid obesity (MO) and normal weight (NW) control subjects.

## Patients and methods

### Study subjects

Patients were recruited from the out-patient clinic for obesity care, University Hospital, Uppsala, Sweden, where preassessments before and long-term check-up visits of patients after surgery were performed. Control subjects were recruited locally. All subjects were White. Exclusion criteria for all three groups were established diabetes or use of glucose-lowering agents, as well as use of ACE-inhibitors, angiotensin II receptor blockers or β-blockers, to avoid possible influence on glucose, proinsulin and insulin status.

Three groups of subjects were compared

- Ten surgically treated patients considered weight stable after weight loss due to GBP surgery, with body mass index (BMI) 34.8 ± 6.2 kg/m^2^ (mean ±sd), who had undergone GBP surgery 1–5 years (mean 4.1 years) before study. The mean presurgery BMI was 45.3 ± 7.6 kg/m^2^. The mean absolute weight loss was 31.8 kg (range 16–47 kg). The mean fall in BMI after surgery was 10.5 kg/m^2^ (23%).
- Ten surgically untreated patients with MO, with BMI 44.0 ± 3.1 kg/m^2^. These patients had refused surgery or were on the waiting list for surgery after preassessment. Their mean BMI was similar to the presurgery BMI in the group of subjects who underwent GBP surgery (*P* for difference = 0.59).
- Twelve healthy NW control subjects, BMI 23.2 ± 2.4 kg/m^2^. Age and sex distribution were similar in the three groups (Table 1).

**Table 1:** Clinical characteristics in morbidly obese subjects, obese subjects after GBP surgery and normal weight control subjects in the fasting state of the study

	Morbidly obese (A)	GBP treated (B)	Control group (C)	Group A vs. B *P*-value	Group B vs. C *P*-value	Group A vs. C *P*-value
Sex (women/men)	5/5	5/5	6/6	—	—	—
Age (years)	41.6 ± 6.8	44.7 ± 5.3	41.1 ± 7.5	0.272	0.218	0.872
Body mass index (kg/m^2^)	44.0 ± 3.1	34.8 ± 6.2	23.2 ± 2.4	0.005	< 0.001	< 0.001
Weight (kg)	133.8 ± 20.2	106.1 ± 28.9	70.9 ± 12.5	0.023	0.001	< 0.001
Height (cm)	174.1 ± 12.1	173.5 ± 13.6	174.2 ± 9.6	0.918	0.882	0.974
f-P-glucose (mmol/l)	5.3 ± 0.6	5.2 ± 0.7	4.8 ± 0.6	0.721	0.225	0.098
HbA_1c_ (%)	4.5 ± 0.3	4.5 ± 0.2	4.2 ± 0.2	0.800	0.018	0.018
f-P-proinsulin (pmol/l)	19.4 ± 18.1	7.0 ± 3.5	5.9 ± 7.4	0.047	0.680	0.028
f-P-insulin (pmol/l)	56.4 ± 28.2	24.6 ± 13.8	19.8 ± 13.2	0.005	0.408	0.001
PIR	0.34	0.28	0.30	0.74	0.33	0.26
f-P-FFA (mmol/l)	0.96 ± 0.32	0.74 ± 0.13	0.78 ± 0.32	0.052	0.716	0.182
f-P-TG (mmol/l)	1.97 ± 1.00	1.37 ± 0.70	0.91 ± 0.65	0.137	0.128	0.007

Data given are arithmetic mean ±sd.

f, Fasting; P, plasma; PIR, proinsulin to insulin ratio; FFA, free fatty acids; TG, triglycerides; GBP, gastric bypass.

The local ethics committee at the Faculty of Medicine approved the study protocol. All patients gave informed consent.

### GBP surgery procedure

Roux-en-Y GBP excludes the stomach and duodenum from the passage of food [18]. The flaccid part of the lesser omentum was opened and the first gastric vessel divided at the lesser curvature, just below the fat pad, to create a small gastric pouch (2 × 3 cm). The opening for the first horizontal stapler was made very close to the wall of the stomach, taking care not damage the nerve-vessel bundle containing the vagal nerve. The pouch was then totally separated from the main stomach, which was left in the abdomen. The small bowel was divided 30 cm distal to the ligament of Treitz and the distal end connected to the small gastric pouch. This jejunal limb, the so-called Roux limb, was made at least 50 cm long and placed behind the excluded stomach and transverse colon. Small bowel continuity was maintained by an entero-enterostomy between the Roux limb and the previously divided proximal jejunum. This created the Y-shaped junction where the ingested food, via the Roux limb, and the gastric acid and bile are mixed. The procedure was done under general anaesthesia, through a short upper-midline incision.

### Test meal

The meal was composed bearing in mind the amount of food it was possible to eat due to the reduced gastric volume after GBP surgery, as follows: a hamburger containing minced beef 75 g, oatmeal 8 g, potato flour 2.5 g, milk 28 g, boiled egg 10 g and onion 5 g; brown sauce 70 g; boiled potatoes 130 g; and carrot 75 g. Dessert consisted of oat bread 40 g with margarine 13 g and raspberry jam 17 g. Participants drank 200 ml of water directly after the meal. The total energy content was 2400 kJ (570 kcal), consisting of: carbohydrates 68.2 g, fat 22.3 g, proteins 24.6 g and fibre 6.4 g.

### Test meal procedures

After an overnight fast for 17 h the standardized test meal was ingested at 13.00 h in the obesity out-patient clinic, allowing supervision of food intake, which was finished within 15 min. Blood samples were collected, centrifuged and freshly frozen immediately before the test meal and thereafter at 30, 60, 90, 120 and 180 min after ingestion. Data were collected on preprinted Case Report Forms.

### Clinical measurements

Weight (kg) and height (m) were measured on standardized, calibrated scales and BMI (kg/m^2^) was calculated. All patients completed a standardized questionnaire, including questions concerning their health status and smoking habits.

### Laboratory analyses

At the laboratory of the Department of Public Health and Caring Sciences/Geriatrics, University Hospital, Uppsala, plasma proinsulin and insulin concentrations were determined using the Proinsulin ELISA and the Insulin ELISA immunoassays (Mercodia AB, Uppsala, Sweden) on a Bio-Rad Coda automated EIA analyser (Bio-Rad Laboratories, Hercules, CA, USA). Concentrations of serum FFA were determined using the Wako NEFA C-test kit (Wako Chemicals GmbH, Neuss, Germany) and serum TG by a lipase and quinoneimine dye method (Konelab) on a Konelab analyser (Thermo Clinical Laboratory Systems Oy, Vantaa, Finland). At the Department of Medical Sciences, University Hospital in Uppsala, plasma glucose concentrations were determined using a routine glucose oxidase technique (Beckman Glucose Analysers; Beckman, Fullerton, CA, USA).

All plasma samples before and after the meal for all subjects were analysed as one batch, thus avoiding drift of determinations.

Basal routine tests for concentrations of HbA_1c_, aspartate aminotransferase, alanine aminotransferase, creatinine, sodium, potassium, haemoglobin and white cell count were analysed using routine methods at the Department of Clinical Chemistry, University Hospital, Uppsala.

### Statistics

**FIGURE 1:**
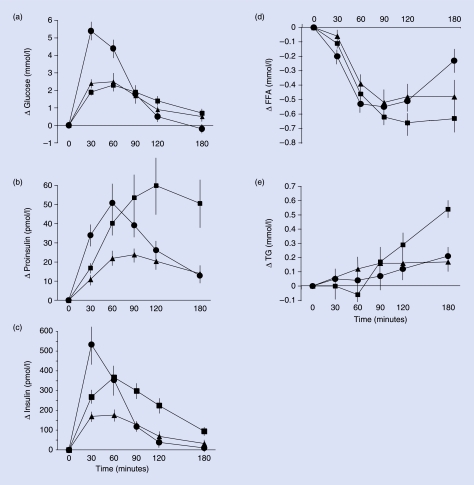
The postprandial changes in glucose (a), proinsulin (b), insulin (c), free fatty acid (FFA) (d) and triglyceride (TG) (e) concentrations are shown for 180 min after the ingestion of the standardized test meal (mean ±sem). 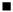, Morbidly obese (MO) subjects; 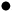, MO subjects treated with gastric bypass (GBP) surgery; 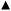, normal weight (NW) control subjects. Glucose: a rapid increase in glucose concentration during the first 30–60 min (early phase) was observed in the GBP-treated group compared with MO subjects (30 min, *P* < 0.001; 60 min, *P* = 0.002) and NW control subjects (30 min, *P* < 0.001; 60 min, *P* = 0.015). In the late phase after ingestion (120–180 min), glucose was significantly lowered in the GBP-treated group compared with MO subjects (120 min, *P* = 0.009; 180 min, *P* = 0.004) and NW control subjects at 180 min (*P =* 0.02). Proinsulin: at all postprandial time points, except at 30 min, proinsulin concentrations were higher in MO than in control subjects (*P =* 0.030–0.008). Early-phase proinsulin concentrations were higher in the GBP-treated group compared with control subjects (30 min, *P* = 0.002; 60 min, *P* = 0.006), but were similar to the MO group. In the late phase, concentrations of plasma proinsulin were significantly lower in the GBP-treated group compared with the MO group (120 min, *P* = 0.048; 180 min, *P* = 0.009), and the GBP-treated group had similar concentrations to control subjects (120 min, *P* = 0.307; 180 min, *P* = 0.814). Insulin: the GBP-treated group had a rapid increase in insulin concentration at 30 min in the early phase that had decreased at 60 min (30 min, *P* = 0.001; 60 min, *P* = 0.026) compared with control subjects. The late-phase insulin response was significantly higher in the MO group compared with the GBP-treated group (120 and 180 min, *P* < 0.001). Insulin concentrations during the late phase in the GBP-treated group did not differ from control subjects (120 min, *P* = 0.072; 180 min, *P* = 0.130). Free fatty acids: no differences were observed regarding postprandial changes between the three groups, except at 180 min, where FFA were higher in the GBP group (*P =* 0.016). Triglycerides: no differences were observed regarding postprandial changes between the three groups, except at 180 min, where TG were higher in the MO group (*P <* 0.001).

All analyses were defined *a priori*. Results are given as arithmetic mean with sd and sem. anova and Students *t*-test were used for group comparisons. Tests were two-tailed and a *P*-value < 0.05 was considered significant. Test meal data are given as absolute concentrations (Table 2) and changes in concentrations (Fig. 1a–e). Statistical analyses were performed using changes from basal concentrations, because absolute differences in basal concentrations of variables (glucose, proinsulin, insulin, TG and FFA) were expected between groups. Areas under the curve (AUC) for glucose, proinsulin, insulin, FFA and TG were also calculated for group comparisons. Statistical software JMP 3.0 for PC (SAS Corp., Cary, NC, USA) was used.

**Table 2:** Postprandial test meal data in morbidly obese subjects, obese subjects after GBP surgery and normal weight controls

	Time points					
	
	0 min	30 min	60 min	90 min	120 min	180 min
MO group						
P-glucose (mmol/l)	5.3 ± 0.6	7.2 ± 0.7	7.6 ± 0.8	7.2 ± 1.0	6.7 ± 0.7	6.0 ± 0.9
P-proinsulin (pmol/l)	19.4 ± 18.1	36.3 ± 23.8	59.7 ± 36.8	73.1 ± 59.6	79.4 ± 64.7	70.1 ± 56.6
P-insulin (pmol/l)	56.4 ± 28.2	325 ± 120	424 ± 92.4	355 ± 145	281 ± 132	151 ± 70.2
P-FFA (mmol/l)	0.96 ± 0.32	0.86 ± 0.31	0.50 ± 0.25	0.35 ± 0.14	0.30 ± 0.08	0.33 ± 0.09
P-TG (mmol/l)	1.97 ± 1.00	1.97 ± 0.96	1.91 ± 0.89	2.14 ± 0.97	2.26 ± 0.99	2.51 ± 1.15
GBP group						
P-glucose (mmol/l)	5.2 ± 0.7	10.6 ± 1.7	9.6 ± 1.9	7.0 ± 1.7	5.7 ± 1.2	5.0 ± 0.9
P-proinsulin (pmol/l)	7.0 ± 3.5	41.0 ± 20.6	57.9 ± 31.0	46.2 ± 22.9	33.2 ± 16.6	19.8 ± 9.7
P-insulin (pmol/l)	24.6 ± 13.8	558 ± 316	379 ± 245	143 ± 69.6	63.0 ± 30.6	34.8 ± 17.4
P-FFA (mmol/l)	0.74 ± 0.13	0.54 ± 0.15	0.20 ± 0.06	0.18 ± 0.04	0.23 ± 0.06	0.50 ± 0.14
P-TG (mmol/l)	1.37 ± 0.70	1.42 ± 0.76	1.41 ± 0.55	1.44 ± 0.62	1.49 ± 0.63	1.59 ± 0.65
NW control group						
P-glucose (mmol/l)	4.8 ± 0.6	7.3 ± 1.0	7.4 ± 1.3	6.5 ± 1.0	5.8 ± 0.7	5.3 ± 0.7
P-proinsulin (pmol/l)	5.9 ± 7.4	17.1 ± 10.8	27.8 ± 14.6	29.7 ± 11.8	26.3 ± 8.9	19.9 ± 10.8
P-insulin (pmol/l)	19.8 ± 13.2	190 ± 91.2	196 ± 97.2	147 ± 67.8	88.8 ± 37.8	52.2 ± 38.4
P-FFA (mmol/l)	0.78 ± 0.32	0.69 ± 0.27	0.38 ± 0.23	0.26 ± 0.11	0.29 ± 0.19	0.29 ± 0.14
P-TG (mmol/l)	0.91 ± 0.65	0.95 ± 0.89	1.03 ± 0.97	1.07 ± 1.02	1.07 ± 0.96	1.08 ± 0.81

Data given are arithmetic means (sd).

P, Plasma; FFA, free fatty acids; TG, triglycerides; MO, morbidly obese; GBP, gastric bypass; NW, normal weight.

A power calculation indicated the need for 10 persons in each group to detect a difference of 20% in changes of proinsulin concentrations, with an α error level of 5% and a power of 80% for a two-tailed Student's *t*-test.

## Results

### Fasting data

Clinical characteristics of the study subjects are presented in Table 1. Fasting glucose concentrations and HbA_1c_ values were within the normal reference ranges in all subjects. The mean fasting plasma glucose concentration was 0.4 mmol/l higher (non-significant) in the GBP group and 0.5 mmol/l higher in the MO group compared with the NW control subjects. HbA_1c_ was 0.3% higher in both the GBP and MO groups compared with NW control subjects (*P <* 0.05 for both).

Fasting proinsulin and insulin did not differ between the GBP and NW control subjects, whereas proinsulin and insulin concentrations were significantly higher in the MO group compared with the GBP and NW control subjects, respectively. The mean fasting FFA value in the MO and GBP groups was not significantly different. TGs were higher in MO group, as expected.

### Postprandial data

Absolute concentrations of glucose, proinsulin, insulin, FFA and TG are shown in Table 2.

### Glucose

The AUC (mean ±sd) for glucose (mmol × min) did not differ between the MO group (270 ± 92), the GBP group (310 ± 110) and NW control subjects (250 ± 130) (*P* for trend = 0.27).

Data for changes in glucose are presented in Fig. 1a. The glucose concentrations were markedly increased during the early phase (0–60 min) in the GBP group (rapid stomach transit) compared with the MO group and NW control subjects. During the intermediate (60–120 min) and the late (120–180 min) phases after ingestion, the reduction in glucose concentrations was larger in the GBP group compared with the MO group and NW control subjects. The MO group and NW control subjects had similar postprandial glucose responses.

### Proinsulin

The AUC (mean ±sd) for proinsulin (pmol × min) was largest in the MO group (7600 ± 5300), intermediate in the GBP group (5300 ± 2700) and lowest in NW control subjects (3000 ± 1600) (*P* for trend = 0.02). Data for changes in proinsulin are shown in Fig. 1b. In the early phase, proinsulin concentrations were higher in the GBP and MO groups compared with NW control subjects. In the intermediate phase, plasma proinsulin began to decrease in the GBP group, but was still increasing in the MO group. In the late phase, concentrations of plasma proinsulin were significantly lower in the GBP group, similar to the NW control subjects, but were still high in the MO group.

### Insulin

The AUC (mean ±sd) for insulin (pmol × min) was highest in the MO group (41 000 ± 9900), intermediate in the GBP group (32 000 ± 16 500) and lowest in NW control subjects (18 000 ± 8300) (*P* for trend < 0.001). Data for changes in insulin are presented in Fig. 1c. The GBP group had a rapid increase in insulin concentrations in the early phase (peak at 30 min) compared with the MO group. Insulin rapidly decreased in the GBP group in the intermediate phase. The late-phase insulin response was similar in the GBP group and NW control subjects. In the MO group the late insulin response was four times higher compared with the GBP group.

### Proinsulin to insulin ratio

The proinsulin to insulin ratio decreased identically in all three groups during the first 30 min. The mean ratio decreased from approximately 0.32–0.10, with no significant difference between groups (*P =* 0.48 and 0.39).

### Free fatty acids

The AUC (mean ±sd) for FFA (mmol × min) did not differ between the MO group (84 ± 31), the GBP group (68 ± 24) and NW control subjects (63 ± 54) (*P* for trend = 0.44).

FFA absolute concentrations, shown in Table 2, were higher (statistically significantly at 30, 60 and 90 min; *P* = 0.03–0.007) at each postprandial time point except at 180 min in the MO group compared with the GBP group and NW control subjects. Prior to 180 min, the FFA values were all lower in the GBP group compared with the NW control subjects and the MO group.

No significant differences were observed regarding postprandial changes in circulating FFA and concentrations between the three groups, except at 180 min, where FFA were higher in the GBP group (Fig. 1d).

### Triglycerides

The AUC (mean ±sd) for TG (mmol × min) was greatest in the MO group (32 ± 29), intermediate in the GBP group (16 ± 22) and lowest in NW control subjects (8 ± 15), although the differences did not reach statistical significance (*P* for trend = 0.07). TG absolute concentrations, presented in Table 2, were significantly higher (*P =* 0.06–0.003) at each postprandial time point in the MO group compared with the GBP group and NW control subjects. No significant differences were observed regarding postprandial changes in circulating TG and concentrations between the three groups, except at 180 min, where TG were higher in the MO group (Fig. 1e).

### Pearson product moment correlation coefficients

BMI correlated with AUC for proinsulin (*r*^2^ = 0.19, *P* = 0.006) and with AUC for insulin (*r*^2^ = 0.35, *P* < 0.001), but not with AUC for glucose (*r*^2^ = 0.03, *P* = 0.34), AUC for FFA (*r*^2^ = –0.03, *P* = 0.87) or AUC for TG (*r*^2^ = 0.06, *P* = 0.15).

### Gender comparisons

Fasting glucose, proinsulin, insulin, FFA and TG did not differ between men and women in the three groups. There were no differences between men and women regarding changes in glucose, proinsulin, insulin, proinsulin to insulin ratio, FFA or TG during the postprandial phases within the three groups.

## Discussion

Postprandial proinsulin concentrations in GBP-treated subjects were lower than in obese subjects, but higher than in normal weight control subjects when expressed as the AUC, but notably proinsulin concentrations were normalized 180 min postload. In the GBP group, a rapid rise in glucose concentrations during the early postprandial phase was followed by a rapid decline ending in even lower concentrations in the later phase than in NW control subjects. The AUCs for glucose over the 180 min studied were similar in all three groups. The rapid emptying of the meal directly into the small intestine without the normal gastric lag time delay may partly explain the pattern of glucose response in the GBP group.

The rapid rise in glucose induced a rapid response, i.e. a rise in proinsulin concentrations in the early phase with a peak at 60 min in the GBP group. The corresponding peak in the MO group was seen at 120 min and in this group postprandial proinsulin concentrations were persistently high throughout the 180-min postload period, reflecting a high demand on pancreatic B-cell secretion.

The high fasting proinsulin concentrations in the MO participants, indicating insulin resistance, were significantly lowered in the GBP group and similar to NW control subjects, indicating improved insulin sensitivity by the induced weight loss in the group treated with bariatric surgery. Weight loss is more pronounced after GBP surgery than after gastric banding [17,19]. GBP surgery also improves the entero-insular axis and glucagon-like peptide-1 secretion [20]. Gastric banding reduces fasting proinsulin concentrations by up to 50%, but does not normalize them [21]. In the present study, markedly lowered proinsulin concentrations were observed in the GBP group, almost similar to those in normal control subjects in the fasting and late phase after ingestion, clearly indicating increased and almost normalized insulin sensitivity and thus markedly reduced B-cell demand.

Previously, insulin concentrations and not proinsulin have been analysed in meal tolerance or glucose tolerance studies of obese subjects or obese subjects who had undergone GBP surgery [22,23]. One pilot study which measured proinsulin concentrations after a test meal in obese compared with normal control subjects was too small to be conclusive [24]. The present study analysed glucose, proinsulin, insulin and the proinsulin to insulin ratio after a standardized test meal where effort was made to compose a test meal representing an ‘every-day lunch’ for GBP-treated subjects. The caloric content was 2400 kJ, which is similar to that of a 75-g oral glucose tolerance test (OGTT). The rapid glucose rise observed in the GBP group induced a rapid insulin response that peaked at 30 min postload, followed by relative hypoinsulinaemia during the latter half of the test. The first 30–60 min of a test meal or an OGTT reflect the first phase of insulin secretion, which has an important role in switching metabolic processes between fasting and postprandial states, mainly by inhibiting hepatic glucose production. A poor or inappropriate first phase of insulin secretion is associated with unsuppressed postprandial glucose production, leading to subsequent higher postprandial glycaemia [25]. Abnormal first-phase insulin secretion is associated with an increased risk of T2DM [26–28] and elevation of the proinsulin to insulin ratio [29], which is also related to T2DM development [30]. In the present study of subjects free of diabetes, the fasting proinsulin to insulin ratios did not differ between groups and decreased similarly after ingestion in all three groups during the early phase, indicating an intact first-phase insulin release, similar to findings using the hyperglycaemic clamp in obese subjects free of diabetes [31].

When studying changes at each time point of the test meal, analysing proinsulin and insulin, we were able to analyse early as well as late responses that were not fully seen when comparing AUCs between groups. The postload AUCs of proinsulin and insulin correlated with fasting concentrations of proinsulin, insulin and BMI, and thus to a great extent reflected the degree of insulin sensitivity. In the MO group, proinsulin concentrations were persistently high during the entire 180-min test. This observation, taking into account the contributory factor of the substantially longer half-life, 115 min for proinsulin compared with 5 min for insulin [32], and that regular meals are often ingested at 3–4-h intervals during the day, might indicate that meal-induced B-cell strain contributes to raised proinsulin throughout a substantial period of the day in obesity. It remains unknown whether such elevated postprandial proinsulin concentrations are associated with increased risk of type T2DM [5–7] or CHD [8–10], as has been reported for fasting concentrations.

In obesity, insulin resistance is common and precedes the progression to impaired glucose tolerance (IGT) and T2DM [33]. Fasting proinsulin concentrations are elevated in the IGT state, but also precede the prediabetic state of IGT [34] and are an independent predictor for T2DM. Surgical treatment with GBP in obese subjects improves insulin sensitivity [14] even though patients are still obese [15]. A novel finding in the present study was the markedly lowered fasting proinsulin and the postprandial proinsulin response that were normalized at 180 min after the test meal, indicating lowered B-cell demand throughout the test and also reflected by the relative hypoinsulinaemia in the late phase postload (Fig. 1c). As expected, fasting and postprandial FFA and TG absolute concentrations were higher in the MO group throughout the test, but a difference in changes of FFA or TG was observed only at 180 min, when FFA changes were highest in the GBP-treated group, possibly due to low inhibition of lipolysis caused by a state of relative hypoinsulinaemia. Lowest absolute values of FFA were observed in the GBP group at all time points up to 120 min because of the insulin peak and secretion, which strongly suppress lipolysis up to this time point.

The relative hypoinsulinaemia in the late phase of the test in the GBP group followed the rapid decrease in both glucose and insulin after peak concentrations in the early phase, and coincided with the time point when symptoms of the so-called dumping syndrome may occur [16]. The mechanisms behind gastric discomfort and fatigue are not fully understood, but the rapid shift in glucose and insulin concentrations, relative hypoinsulinaemia and hypoglycaemia may contribute to such symptoms [16,35].

In summary, GBP surgery results in dramatically improved insulin sensitivity and rapid meal-stimulated secretions of proinsulin and insulin with sustained effects on glucose and lipid metabolism. Our results suggest that future studies should determine whether proinsulin is a marker for diabetes risk before and after GBP surgery. It might be speculated that the proinsulin status can be used as an indication for bariatric surgery. To answer such questions, further research on proinsulin and insulin as markers for T2DM and CHD in long-term follow-up after GBP surgery is warranted.

## Competing interests

None to declare.

## Abbreviations

BMI: body mass index
CHD: coronary heart disease
FFA: free fatty acids
GBP: gastric bypass
IGT: impaired glucose tolerance
MO: morbid obesity
NW: normal weight
OGTT: oral glucose tolerance test
T2DM: Type 2 diabetes mellitus
TG: triglycerides
